# The Re-Introduction of Small-Pox.—I

**Published:** 1898-08-27

**Authors:** 


### Modern Sociology.

THE RE-INTRODUCTION OF SMALL-POX.—I.

On many occasions in history, both in ancient and modern times, it has happened that the supineness or malfeasance of rulers has brought about evils with regard to which the neglected or abandoned provisions of the law have been supplied, perhaps in an irregular manner, perhaps at the cost of undue suffering to some of those concerned, but still, in the main, successfully. In such circumstances, it may often be expedient for the unprotected to combine together for the purpose of supplying the defects of the law, and thus, by united action, to avoid the assumption of any individual responsibility for departing from it.

At the present time, as a direct consequence of the action of the Government, we are threatened with a recurrence of small-pox under conditions which can hardly fail to be productive of the epidemic diffusion of the disease; and it is, therefore, worth while to consider what this diffusion would imply, and what measures, if any, can be taken by the general public for the purpose of protecting themselves and their children.

It must, in the first place, be remembered that, from causes which are not clearly understood, the continued prevalence in a population of any microbial disease appears to confer, not indeed immunity, but at least some increased measure of resisting power; so that, while such a disease may be only severe and formidable in a country in which it has long been present, it becomes a destructive pestilence in any into which it is freshly introduced. To small-pox this general principle has a peculiar applicability. The disease seems to have been brought to England and Southern Europe by returning Crusaders, and it prevailed extensively in these localities from the ninth to the sixteenth centuries, destroying many lives, producing much blindness, and permanently ruining the health of thousands of those whom at first it failed to kill. In 1517 it was carried from Europe to St. Domingo, and from thence to Mexico, where it is said to have destroyed three millions and a half of people. In 1707 it appeared for the first time in Iceland, and killed more than a fourth part of the inhabitants; and in 1733 it reached Greenland, and almost depopulated the country. With regard to the degree of its ordinary infectiveness, Dr. Haygarth, the originator of isolation and of special hospitals for infectious diseases, wrote, in 1793, that, during his long attention to the subject, he had never known an instance of a susceptible person being in the same room with a small-pox patient without contracting the disease; and the experience of France, during the war of 1870-71, showed that in this respect the character of the malady had undergone no mitigation. The same lesson has been taught still more recently by the epidemics at Willenhall and at Gloucester. At the last-mentioned place, with a population of 42,000, among whom scarcely any children of the poorer classes had been vaccinated for ten years before, a few dropping cases appeared towards the end of 1895. In the first half of 1896 the cases amounted to 2,000, and there were 443 deaths, of which 285 were in children under ten. In the month of April there were 744 fresh cases, but by that time the local authorities had taken fright at the consequences of their past neglect, and there was a rush for vaccination. Thirty-six thousand persons were vaccinated or re-vaccinated, and the plague was stayed. By the arrival of July it was over, but the president of the local chamber of commerce has shown that it cost the inhabitants, at a very moderate estimate, not less than £150,000.

The events which occurred in Gloucester are likely, in the course of the next few years, to be repeated in many other places. Ignorant people, that is to say, the great bulk of the population, have no conception of what the prevalence of small-pox implies. They have no experience of conditions which were familiar a century ago, and they have not sufficient imagination to be able to realise those conditions from descriptions, even if the descriptions came in their way. They will be told that vaccination is no longer enforced by law, and they will infer that the wisdom of the Legislature has discovered that the operation is either ineffective or superfluous. Vast numbers of children will be left unprotected; and vast numbers more will be protected but imperfectly. Tender mothers will perhaps so far yield to medical advice and persuasion as to consent to the operation, but on condition that only one arm shall be "done," or that not more than two punctures shall be made. The immunity conferred by vaccination is now known to be both less complete and less enduring than Jenner hoped and believed it would be, and has been shown to fall strictly into line with the forms of immunity conferred by analogous proceedings against other diseases. It can only be rendered complete by the fulfilment of certain conditions with regard to the number and the course of the vesicles; and it can only be rendered permanent by repetition. This being so, an outbreak of small-pox in any populous place is likely to find a large population of fully susceptible people, and another large population of people who, although not fully susceptible, will be liable to contract the disease in a mild or modified form, and to communicate it to others in its greatest virulence. The infectiveness of small-pox is well known to be enormously increased by the aggregation of cases; insomuch that every small-pox hospital, even under the existing conditions of general vaccination, has been found to diffuse the disease over a considerable area; which, when examined in concentric circular belts, has yielded cases in strict numerical ratio to the distance of each belt from the centre. Neither in these nor in any other conditions is any danger of small-pox incurred by those who have been properly vaccinated and re-vaccinated; but such persons, even if free from risk of direct injury, are still citizens, and are, or should be, interested in the welfare of the community to which they belong: while it is certain that they would have no exemption from the losses, in the way of destruction of trade and enhanced expenses of local government, which every prevalence of small-pox must entail. We shall endeavour, in a subsequent article, to show something of the effects of the disease upon the health and appearance of those whom it fails to kill; and also to point out the lines on which the protected might most usefully organise a system of resistance to the spread of future epidemics.

